# The effectiveness of a modified Gui Zhi Fu Ling Wan formulation (Gynoclear™) for the treatment of endometriosis: a study protocol for a placebo-controlled, double-blind, randomised controlled trial

**DOI:** 10.1186/s13063-021-05265-x

**Published:** 2021-04-21

**Authors:** Mike Armour, Mahmoud A. Al-Dabbas, Carolyn Ee, Caroline A. Smith, Jane Ussher, Susan Arentz, Kenny Lawson, Jason Abbott

**Affiliations:** 1 NICM Health Research Institute, Western Sydney University, Locked Bag 1797, Penrith, NSW 2751 Australia; 2 Translational Health Research Institute (THRI), Western Sydney University, Locked Bag 1797, Penrith, NSW 2751 Australia; 3 University of New South Wales, Royal Hospital for Women, Randwick, NSW 2031 Australia

**Keywords:** Endometriosis, Gynoclear, Chinese herbal medicine, Pelvic pain

## Abstract

**Background:**

Endometriosis is the presence of tissue similar to that of the endometrium outside the uterine cavity and is the most common cause of chronic pelvic pain. Current non-surgical treatments such as non-steroidal anti-inflammatories, oral contraceptive pills and hormonal treatments have limited effectiveness, and the side effect profile is bothersome. This study will evaluate the efficacy of Gynoclear™ by change in endometriosis-related pain based on the Endometriosis Pain Daily Diary (EPPD) scores.

**Methods:**

This randomised, double-blind, placebo-controlled trial will recruit a minimum of 90 adult participants across Australia who have a laparoscopic visualisation/confirmation of endometriosis in the last 5 years and have current moderate or greater pelvic pain. Participants will be randomly allocated in a 1:1 ratio to receive either Gynoclear™ (active) or placebo. Gyncolear’s active ingredients are *Carthamus tinctorius* (Safflower), *Cinnamomum cassia* (Chinese cinnamon), *Poria cocos* (Hoelen), *Paeonia suffriticosa* (Tree peony), *Paeonia lactiflora* (Peony) and *Salvia miltiorrhiza* (Red sage). Participants are asked to complete a total of 5 months’ worth of pain diary entries via the EPDD v3, including 1-month screening, 2-month treatment period and 1-month post-treatment follow-up. The primary outcome variable is change in endometriosis-related pain based on the EPDD v3 scores. Secondary outcomes include change in health-related quality of life via the Endometriosis Health Profile (EHP-30), SF-12 and EQ-5D scores as well as changes in rescue analgesic usage, dyspareunia and fatigue via the EPDD.

**Discussion:**

This study will determine the safety and efficacy of Gynoclear™ to reduce the severity and duration of non-cyclical pelvic pain, dysmenorrhoea, dyspareunia and other symptoms of endometriosis. Study outcomes will be of interest to health professionals and members of the public who suffer from endometriosis.

**Trial registration:**

Australia and New Zealand Clinical Trials Registry ACTRN12619000807156. Registered on 3 June 2019.

**Supplementary Information:**

The online version contains supplementary material available at 10.1186/s13063-021-05265-x.

## Background

Chronic pelvic pain (CPP) is pain in the pelvis of greater than 6 months duration, which is severe enough to cause functional disability or require medical intervention [1]. Chronic pelvic pain has a number of aetiologies including endometriosis, adenomyosis, chronic pelvic infection and functional disorders such as irritable bowel syndrome or interstitial cystitis. Endometriosis is the presence of tissue similar to that of the endometrium outside the uterine cavity [2] and is the most common cause of CPP [3].

Worldwide prevalence rates of all types of chronic pelvic pain range from 5.7 to 26.6% [4]. Accurate prevalence rates for endometriosis are difficult to estimate. Reports from clinical settings suggest a rate of around 10% globally [5], and in Australia, around 11% of women aged 40–44 have a diagnosis of endometriosis [6].

Current non-surgical treatments such as non-steroidal anti-inflammatories, oral contraceptive pills and hormonal treatments have limited effectiveness [7], and the side effect profile is bothersome, with discontinuation rates of between 25 and 50% [8]. These factors may contribute to the observation that non-pharmacological self-care use is very common with 75% of Australian women with endometriosis reporting using self-care in the past 6 months. Sixteen percent of these women were using herbal medicine to help manage their endometriosis symptoms or the side effects of their conventional medications [9].

Gui Zhi Fu Ling Wan (GZFLW), a traditional Chinese medicine (TCM), has been used for gynaecological disorders since the fifteenth century [10]. Gui Zhu Fu Ling Wan consists of five herbs, Gui Zhi (*Ramulus cinnamomi*), Fu Ling (*Poria cocos*), Mu Dan Pi (*Cortex moutan radix*), Bai Shao (*Radix Paeoniae alba*) and Tao Ren (*Semen persicae*). The formulation is indicated for chronic pelvic pain, fibroids and severe dysmenorrhoea [11]. A modification of GZFLW, containing similar western herbal species and with the addition of another herb Dan Shen (*Salvia miltiorrhiza*) and the substitution of Tao Ren for another similar herb Hong Hua (*Carthamus tinctorius*), has been used by our partners Metagenics to create Gynoclear™. This particular combination has not been scientifically evaluated for the treatment of endometriosis-related pain.

The aim of this study is to evaluate the efficacy and safety of Gynoclear™ on pelvic pain, fatigue and other quality of life measures in women with a confirmed diagnosis of endometriosis.

## Methods

A randomised, placebo-controlled, double-blind, clinical trial to evaluate the efficacy of a modified Gui Zhi Fu Ling Wan formulation (Gynoclear™) for the treatment of endometriosis-related pelvic pain. Australian ethics approval from Western Sydney University Human Research Ethics Committee, H13256 (approved May 2019). The trial was prospectively registered with the Australia New Zealand Clinical Trials Registry: ACTRN12619000807156. Recruitment began in June 2019 and is currently ongoing. Single centre study recruiting from all of Australia (NSW, VIC, WA, TAS, QLD and SA). Coordinating centre: NICM Health Research Institute at Western Sydney University, Penrith NSW 2751 Australia.

### Participants

Eligible participants are aged 18 to 45, have a laparoscopic visualisation/confirmation of endometriosis in the last 5 years, self-reported menstrual or non-menstrual pelvic pain and at least one of the common endometriosis-related forms of pelvic pain will be recruited. Full inclusion and exclusion criteria are presented in Table 1.

**Table 1:** Inclusion and exclusion criteria

**Inclusion criteria:**	
Age 18–45 years.	
Laparoscopic visualisation/confirmation of endometriosis in the last 5 years.	
Have menstrual or non-menstrual pelvic pain rated ≥4/10 on a numeric rating scale based on an average over 1 month via endometriosis pain daily diary (version 3) scores.	
Report at least ONE of the following:	
Dysmenorrhoea (period pain),	
Dyspareunia (pain during or after sexual intercourse),	
Dyschezia (pain before or during bowel motion) OR	
Dysuria (pain prior to or during urination).	
Willing to provide informed consent and adhere to the protocol.	
Able to travel to a Laverty Pathology (or its sister companies) collection centre for two blood tests; one at baseline and the second at the end of the intervention approximately 12 weeks later	
If sexually active, agreeing to use appropriate contraception to prevent pregnancy during the study period.	
Has internet access (either via a mobile, tablet or computer) for completing the Endometriosis Pain Daily Diary v3 scores.	
**Exclusion criteria:**	
Have had endometriosis related surgery in the previous 6 months or have any surgery planned during the study period.	
Started or stopped/removed any hormonal contraceptive within the last 6 months. This includes the oral contraceptive pill (GnRH-a or danazol), implant contraceptives and the Mirena.	
Started, stopped or changed dosage on any pharmaceutical medication or herbal/natural medicine targeting endometriosis symptoms (such as pregabalin, Nortriptyline, or other Chinese herbal medicine) in the previous 6 months	
Having a known allergy or intolerance to any of the ingredients in Gynoclear™	
Participant has previously used Gynoclear™ for any condition.	
Participant is currently using a herbal supplement that contains any of the main constituents of the Gynoclear™ formula (e.g. Mediherb Endofem).	
Usage of anticoagulants (e.g. Warfarin, Heparin, Eliquis, Pradaxa, Xarelto) or any other medication, including nutritional and/or herbal supplements that may cause blood thinning (e.g. Vitamin E, *Gingko biloba*).	
History of coagulation disorders.	
Currently pregnant or breast feeding or planning on becoming pregnant during the study period.	

Based on a 20% difference in pain (measured by a 0–10 NRS) between groups (a moderate effect size *d* = 0.5 and a clinically significant difference), a standard deviation in pain scores of 2.2 (based on pilot data), an alpha of 0.05 and a power of 80% would mean 74 participants would need to be recruited. Given a predicted 20% drop out rate, 90 participants in total (45 per group) will be recruited. At the time of manuscript submission, 24 participants have been randomised.

### Patient and public involvement

Two streams of focus groups were conducted to co-design the trial with key stakeholders. The two groups were women diagnosed with endometriosis (*n* = 67) and TCM, herbalist or naturopathic practitioners (*n* = 8). These groups provided input on feasibility, dosage, outcome measures and duration of the trial.

### Recruitment and selection

Recruitment for this study is primarily via our partners Endometriosis Australia social media presence as well as paid Facebook advertising. All advertisements will advise participants of the trial and refer them to the Research Institute webpage. A link to a pre-screening survey will be available on the webpage to allow participants to self-screen against the inclusion and exclusion criteria of the study. All potentially eligible participants, based on their responses from the survey, will be instructed to schedule a call with the clinical trial officer or another member of the research team for follow-up procedures.

Potential participants will be required, after giving consent, to fill in a previously validated endometriosis daily pain diary (EPDD v3) [12] via an online secure electronic case report form hosted on Castor EDC. This diary will be filled in for 4 weeks and will fulfil two purposes:

1. Ensure that the participant meets the minimum pain requirements to enter the study and
2. If the participant is included in the study, this becomes the baseline/pre-treatment data for future analysis.

If eligible based on pain diary scores (averaged across 4 weeks), participants will be required to provide a copy of their most recent laparoscopy report and/or the gynaecologists report/letter that confirms visualisation of endometriosis. This must be within the previous 5 years of their entry into the trial. Upon receipt of the completed EPDD and the proof of surgical confirmation, participants will be included in the study.

### Randomisation

After signing the informed consent document and satisfying the eligibility criteria, participants will be randomised in a 1:1 ratio to either the active treatment group (Gynoclear™) or the placebo control.

Using Castor EDC’s randomisation function, a randomisation sequence using a block size of 6, with 1:1 group allocation, was performed on the 15th of January 2019 by NICMs Clinical Trial Manager who is external to this study. Randomisation numbers were allocated in permuted blocks of 6 containing 3 active and 3 placebo randomisation numbers. The investigator will allocate each randomisation number in order of number sequence starting with the lowest number in each block and using all numbers in a block of 6 before starting with the lowest number in the next block of numbers. As soon as the randomisation number is assigned, it will be recorded on the participant log, in the patient notes and case report form. Details of any patients randomised out of sequence will be notified immediately to the Chief Investigator.

### Blinding

All study teams, participants and data analysts are blind to group allocation. Unblinding of participants to the study’s medical monitor, Dr Ee will be permitted if serious adverse events are reported.

### Study interventions

Production of study supplement for the trial will be from one production batch lot and was manufactured according to Good Medical Practice guidelines. Study supplement dosing is six capsules (active or placebo) per day, taken three times daily, two capsules per time, preferably with food. The placebo matches Gynoclear™ (active) in colour, taste and smell. Table 2 outlines the composition of both the interventional product and placebo.

**Table 2:** Composition of interventional product (Gynoclear™)

Active ingredients per capsule (extracts equivalent to)	
*Cinnamomum cassia* twig bark, dry (Cinnamon) 920 mg	
*Poria cocos* fruiting body (Poria) 920 mg	
*Carthamus tinctorius* flower, dry (Safflower) 920 mg	
*Paeonia suffruticosa* root bark, dry (Tree peony) 920 mg	
*Paeonia lactifora* root, dry (Peony) 920 mg	
*Salvia miltiorrhiza* root and rhizome, dry (Red sage) 900 mg	

### Composition of placebo

**Table:** 

Inactive placebo based on cellulose with a non-therapeutic input of cocoa powder (for colour matching).	

### Outcome measures and data capture

All data will be captured electronically via the secure electronic data capture platform Castor EDC [13]. Signed consent forms will be digitally captured and securely stored in Castor EDC.

Participants will not be required to physically attend any of the study visits for the trial, with the exception of two blood tests at certified pathology collection centre. The total study duration is 20 weeks: 4 weeks prior to trial entry to perform baseline pain screening diary, 12 weeks of active treatment and 4 weeks of follow-up. Six study visits/checkpoints will be required: (1) screening (confirm eligibility), (2) baseline (blood collection followed by dispensing medication), (3) week 6 phone call (check adverse events, compliance and medication use), (4) midpoint (week 12; check adverse events, compliance, medication use and dispense medication), (5) end of treatment (week 16; check adverse events, compliance and medication use and blood collection) and (6) post-treatment follow-up (week 20; check adverse events and collect pain diary scores of 1 month).

All data will be securely held on the study team database, accessible only by authorised investigators for the purposes of monitoring. The final dataset will be accessible by the coordinating investigators and each locality will retain on-site access to digital copies of source documentations with paper copies stored in secure archives.

Any changes to the study protocol are provided to the Human Research Ethics Committee as per protocol, trial registries will be updated. A copy of the consent form is included as Supplementary File 1.

Clinical assessments and patient surveys will be completed using Castor clinical trials management software. All the pre-specified outcome measures are outlined in Table 3:

**Table 3:** Outcome measures

**Primary outcome measure:**	
To evaluate the efficacy of Gynoclear™ by change in endometriosis-related pain based on the Endometriosis pain daily diary v3 (EPDD) scores.	
**Secondary outcome measures:**	
To assess the change in health-related quality of life via the Endometriosis Health Profile (EHP-30), SF-12 and EQ. 5D scores.	
To assess any changes in use of pharmaceutical analgesics via the EPDD	
To assess any changes in dyspareunia (painful sexual intercourse) via the EPDD.	
To assess any changes in fatigue via the EPDD and fatigue severity scale (FSS)	
To assess any changes in restrictions to activities of daily living via the EPDD.	
To monitor the frequency and severity of adverse events during intervention period.	
To determine the cost-effectiveness of using Gynoclear™.	
To explore participant satisfaction with the intervention.	

#### EPDD

Participants will have access to the EPDD v3 [12] via an online web form. After focus groups with over 40 women with endometriosis, they identified that their top three symptom priorities were pelvic pain, fatigue and dyspareunia. The EPDD v3 already tracks pelvic pain and dyspareunia so modifications were made to include a daily fatigue score (0–10) in the same format.

#### Participant expectation and satisfaction questionnaires

At trial entry, participants will be asked about their current symptoms, and what expectations they have regarding changes of pain and other symptoms during the trial.

At the trial exit, participants will be asked to indicate which group they thought they were in, rate their satisfaction with the treatment given, what (if any) symptoms changed and what impact this had on them. Additional open-ended questions will explore acceptability including their experiences participating in the trial, likelihood of recommendation of the intervention to family and friends, interest in using the intervention again for pelvic pain symptoms, and feedback on the trial design and outcomes collected.

#### Health-related quality of life questionnaires

The Endometriosis Health Profile-30 (EHP-30) is an endometriosis specific health-related quality of life measure [14]. It covers pain, control and powerlessness, social support, emotional well-being and self-image. These 30 questions provide the core of the EHP-30. The EHP also includes optional secondary modules that may not be appropriate to all participants. If participants are currently working, they will be asked to fill in the ‘Work’ module, if they are currently in a sexual relationship, they will be asked to fill in the ‘Sexual relationship’ module. All measures have a 1 month recall and will be taken twice online; at baseline and at the end of the 4-week follow-up period.

The EQ-5D [15] and SF12 [16] are validated health-related outcome measures and provide the necessary data for economic analysis and have been recently used in our economic analysis of the impact of endometriosis in Australia [17]. The EQ-5D is administered via Castor EDC and the SF-12 will be administered via paper-based methods due to cost, at baseline and at the end of the 4-week follow-up period.

#### Fatigue severity scale (FSS)

The fatigue severity scale is a nine-item, seven-point questionnaire used to determine the impact of fatigue when performing daily activities [18]. This will be used as an adjunct to the numerical ratings given for daily fatigue, to provide a measure of the real-world impact of fatigue. This tool uses a seven-day recall. This will be administered online at baseline and at the end of the intervention.

### Participant safety

The product has been listed as a listed medicine on the Australian Register of Therapeutic Goods (ARTG) (AUST L 197899). As the investigational product (Gynoclear™) is a low-risk product already approved by the Australian Therapeutic Goods Administration and available for purchase through health care practitioners. No adverse effects from oral consumption at the dosages outlined have been reported to the Australian Therapeutic Goods Administration. A formal data monitoring committee will not be established in Australia, however, the nominated medical representatives for the study, Dr Carolyn Ee, is a registered General Practitioner in Australia and will review safety on all adverse events.

Methods for adverse event recording and reporting include at all study visits beginning at the week 2 phone call and up until trial completion. Participants are also encouraged to report any safety concerns as soon as they become aware of it and have been provided with a written information sheet detailing the research team’s contact details (i.e. Clinical Trial Coordinator and Chief Investigator) for referral to the Dr Ee.

Safety markers in the blood will be tested at baseline and at trial exit. Participants will be asked to go to their local Laverty Pathology (or sister companies depending on region) collection centre. These safety tests will consist of the following markers in the blood: liver enzymes, bilirubin and albumin (liver function tests (LFTs)), urea and electrolytes (U&E) and red and white blood cells (full blood count). Electronic copies of blood test results will be sent to the medical representative of this study (Dr Carolyn Ee) for review when there are indications that blood markers are outside normal reference ranges.

### Compliance

At each contact point (2-week phone call and midpoint call) compliance is checked verbally. At the midpoint, participants will be asked to return their three most empty trial product containers. These will be checked for compliance (75% or greater adhereance to the daily dose will be considered compliant) before the remaining trial product is dispensed. A similar procedure will occur at the end of treatment where participants are asked to return all remaining trial product.

### Withdrawal

Participants who withdraw will have the reason for withdrawal (e.g adverse events, lack of efficacy) documented.

### Concomitant medication

All participants are advised to continue taking their medications as per their doctors’ advice. Changes in concomitant medication are monitored via the study diaries.

### Analysis plan

All data is captured via Castor EDC digitally, there are no paper instruments used in this trial. Data will be exported from Castor EDC into SPSS v24 (or greater). Prior to analysis, data will be cleaned by examining frequencies, means, medians, and ranges to identify logical errors. Instruments will be coded according to their respective scoring instructions. Unless specified by the instrument, missing items will be imputed based on averaging values within the instrument for a given individual, if no more than 15% of items are missing. Otherwise, that instrument will be set to missing for the individual.

Baseline demographics will be reported using descriptive statistics. Daily pain scores (as measured by the EPDD) will be converted into a single pain score at five time points, baseline, month 1, month 2, trial exit/end of intervention and follow-up. This single pain score will be achieved by taking the mean of the daily pain scores for the previous 4 weeks. Both a per-protocol and intention to treat analysis will be undertaken for the primary outcome of changes in pain scores. The same process of generating a single score will be used for the following outcomes: pain interference with activities of daily living (0–10), use of analgesics (number of days per month needing additional medication), severity of fatigue (0–10) and severity of dyspareunia (0–10). All scores will be analysed using repeated measures at baseline, month 1, month 2, trial exit and follow-up via a longitudinal linear mixed model analysis of variance with time and group as fixed effects and subject as a random effect.

Secondary outcomes of changes in EHP-30 scores, SF-12 scores and EQ-5D scores will be analysed using analysis of variance between baseline and one-month follow-up. FSS scores will be analysed using paired analysis of variance between baseline and the end of the intervention. Baseline values with be used as covariates in the analysis.

A cost-effectiveness (cost-utility) analysis will be conducted alongside the trial following international best practice [19]. This will assess the difference between trial arms in (i) costs—the cost of introducing modified GZFLW and the use of analgesic medications, measured using Medicare Benefits Scheme/Pharmaceutical Benefits Scheme, and (ii) effects—the difference in an economic measure of health-related quality of life, called the EQ-5D, which is used in estimating quality-adjusted life years. A cost-effectiveness analysis, combining i and ii, will then be conducted including subgroup analysis. Further, statistical uncertainty will be explored in a Probabilistic Sensitivity Analysis, health-related quality of life will be cross-validated using the SF12, and a Value of Information analysis will assess whether further research is required before making recommendations to mainstream modified GZFLW in routine practice.

### Data monitoring and stopping guidelines

Data will be monitored by MaD and MA for completeness, plausibility and consistency to ensure the integrity and completeness of the data set. Any queries will be resolved by the Chief Investigator or delegated member of the study team. Adverse events will be regularly monitored via the online daily diary, as well as at each study visit conducted over the phone by the research team. Any serious adverse events will trigger an alert to the Chief Investigator and reporting to relevant authorities as per the National Health and Medical Research Council guidelines.

### Study timeline

The expected duration of the data collection phase of this study will be 12 months, with 14 time points where data is collected. The schedule of enrolment, interventions and assessments as per SPIRIT [20] is outlined in Table 4.

**Table 4:** Timeline of treatment assessments and interventions

Procedure	Screening (week 0)	Baseline (week 4)	Phone call (week 6)	Midpoint (week 10)	End of treatment (week 16)	Post-treatment follow-up (week 20)
Informed consent	X					
Inclusion and exclusion	X					
Medical history	X					
Screening blood test: LFT and U&E		X				
Randomisation		X				
Treatment/placebo dispensed		X				
End of treatment blood test: LFT and U&E					X	
Concomitant medication collection		X	X	X	X	X
Participants electronically fill out Endometriosis Pain Daily Diary v3 Scores	X	X	X	X	X	X
Quality of life forms (SF-12, EQ-5D, EHP-30)		X				X
Fatigue severity scale (FSS)		X			X	
Participant expectation and satisfaction questionnaire		X			X	
Adverse events			X	X	X	X
Dispense/return study drug		X		X	X	

### Dissemination of findings

A lay summary of the findings will be provided to all relevant endometriosis support and advocacy groups in Australia and via articles through organisations such as The Conversation. Dissemination through the academy will be via peer-reviewed publications in appropriate journals and at scientific conferences.

### Data sharing plan

After the completion of the study, data will be made available to researchers upon review and approval of the submitted protocols by the research team. The full trial protocol is available via the principal investigator.

## Discussion

Herbal medicine is very commonly recommended by natural health practitioners in Australia and New Zealand, including naturopaths [21] and Chinese medicine practitioners [22], to treat the symptoms of endometriosis. Gui Zhi Fu Ling Wan is the most common Traditional Chinese herbal medicine prescription used in the treatment of endometriosis [23] and includes ingredients commonly recommended by naturopaths and western herbalists [24]. However, the evidence of effectiveness for herbal medicine is limited. Systematic reviews of studies investigating Chinese herbal medicine for endometriosis have been inconclusive due to a lack of high-quality trials [25] and a previous study using raw herbs made into a decoction had issues with finding an inert placebo [26]. While many practitioners use variable complex herbal formulations as part of their treatment protocols [27], the convenience of an encapsulated ‘off-the-shelf’ formula, that can be dispensed through pharmacy and herbal medicine dispensaries may increase the availability of herbal medicine to women with endometriosis.

The economic burden of endometriosis is similar to or higher than other chronic disease burdens such as heart disease and diabetes [28]. Our team has recently completed a study on the cost of illness burden of endometriosis in Australia and found that the total cost per woman per year is $31,137AUD [17]. Our research also examined the cost per woman based on their reported pain score and found that each reduction in pain scores by 20% reduced costs by a minimum of $9000 per woman per year. Therefore, effective pain management strategies are vital to reduce the economic disease burden and improve quality of life. Given that Australian women are already seeking out non-pharmacological treatment for endometriosis [9, 21], if Gynoclear™ is found to be an effective treatment to help reduce endometriosis-related pelvic pain and other associated symptoms of endometriosis, this could be an effective adjunct treatment for the more than 720,000 women with endometriosis in Australia.

Potential limitation to the trial that must be acknowledged is that all outcome measures are self-reported. This is due to the lack of reliable, non-invasive objective outcome measures currently available to track the progression or response to treatment [29]. However, these self-reported measures are in line with the recently developed core outcome set developed by a team of international experts, which do not currently recommend any objective outcome measures unless tracking fertility [30]. Other potential limitations are COVID-19 related, where the requirement for safety blood tests may impact some women’s decision to participate, potentially due to concerns about infection or limitations on travel due to lockdown or social distancing measures.

## Trial status

Protocol Version and Date: v6 23 Oct 2019

Date Recruitment Commenced: 01 Jun 2019

Expected Date of Completion: 31 Jul 2021

## Supplementary Information

**Additional file 1.**

## Data Availability

Not applicable.

## Abbreviations

EPDD: Endometriosis Pain Daily Diary
EHP-30: Endometriosis Health Profile
SF-12: Short Form Health Survey
CPP: Chronic pelvic pain
GZFLW: Gui Zhi Fu Ling Wan
TCM: Traditional Chinese medicine
FSS: Fatigue severity scale
U&E: Urea and electrolytes
LFT: Liver function tests
AUD: Australian dollars
