# Supplementary material for: The effectiveness of a modified Gui Zhi Fu Ling Wan formulation (Gynoclear™) for the treatment of endometriosis: a study protocol for a placebo-controlled, double-blind, randomised controlled trial
Source: Trials. 2021 Apr 21;22:299. doi: 10.1186/s13063-021-05265-x (PMC8058744; doi:10.1186/s13063-021-05265-x)
Supplement: Supplementary file 1 — Additional file 1. [file 13063_2021_5265_MOESM1_ESM.pdf]

WESTERN SYDNEY UNIVERSITY

Participant Information Sheet

Project Title: A placebo controlled, double blind, randomised controlled trial of a modified Gui Zhi Fu Ling Wan formulation (Gynoclear™) for the treatment of endometriosis.

Project Summary:

You are invited to participate in a research study being conducted by Dr Mike Armour, Dr Carolyn Ee, Professor Caroline Smith, Dr Susan Arentz and Mahmoud Al-Dabbas (Clinical Trial Officer) from NICM Health Research Institute, Professor Jane Ussher and Associate Professor Kenny Lawson from Translational Health Research Institute (THRI) at Western Sydney University and Professor Jason Abbott from University of New South Wales. The research involves participating in a clinical trial that will test a modified traditional Chinese herbal medicine (Gynoclear™) for endometriosis related pain and fatigue.

If you are aged 18-45 years, live in Australia, have at least moderate pelvic pain, and have had surgical confirmation of endometriosis in the last five years, you may be eligible to participate in the study. To confirm your eligibility, we will need to ask you more questions before you start.

How is the study being paid for?

This project is funded through a partnership grant between Metagenics, the manufacturer for Gynoclear™, and Western Sydney University.

Why is this study being done?

Endometriosis is the presence of tissue similar to that of the endometrium outside the uterine cavity and is the most common cause of chronic pelvic pain in women. Current non-surgical treatments such as non-steroidal anti-inflammatories, oral contraceptive pills and hormonal treatments have limited effectiveness and the side effect profile is bothersome for many women.

A traditional Chinese medicine called Gui Zhi Fu Ling Wan has been used for the symptoms of pelvic pain since the 15th century and is commonly used in Taiwan and China for the treatment of endometriosis. Gynoclear™ is a modification of Gui Zhi Fu Ling Wan and consists of six herbs (Gui Zhi [*Ramulus Cinnamomi*], Fu Ling [*Poria Cocos*], Mu Dan Pi [*Cortex Moutan Radicis*], Bai Shao [*Radix Paeoniae Alba*], Hong Hua [*Flos Carthami*] and Dan Shen [*Salvia miltiorrhiza]* that are used in Chinese herbal medicine to reduce pain associated with menstruation. This combination could potentially reduce the severity of endometriosis related pain and fatigue. This study will examine if the use of this modified Chinese herbal medicine, compared to a placebo (a treatment that looks the same but doesn't contain any active ingredients), can provide a reduction in pain and fatigue that is related to your endometriosis.

What will I be asked to do?

In summary: After initial assessment and consent, you will be asked to complete an online diary once a day for 4 weeks to confirm your eligibility, as well as some questions about you (demographics) and about your quality of life, and finally to have a blood test for safety checks (your blood count, liver and kidney function). Once this is complete if you are eligible, you will be enrolled in the study. You will then be asked to take six capsules per day for three months and fill in a short online diary form once a day, and at the end of the three months have another blood test for safety checks (your blood count, liver and kidney function). One month after you took your last capsule, we will ask you fill in some final

forms on how you are feeling. Apart from the blood tests, all measurements are either done online, over the phone or by return post.

In more detail:

- Screening (2-3 minutes per day): after an initial eligibility screening and your written consent to participate in the study, you will be asked to fill out a total of 30 days (or 1 months' worth) of an online and secure pain daily diary to assess what your current level of endometriosis related pain. This can be done on your smartphone, tablet or computer.
- Baseline (30 minutes): after another round of eligibility assessment has been performed based off your daily diary responses from the screening visit, you will be asked to have a blood test taken for safety checks (your blood count, liver and kidney function) at a pathology laboratory located near you and this will act as the final determinant of your eligibility for the study. Once your eligibility has been confirmed, you will be randomly allocated to be either in a treatment or placebo group. The research team will then organise for 8-weeks' worth of study medication to be delivered to your home address (or your preferred location) where you will start to take the study medication as per the instructions given to you from the research team. You will also be asked to complete three separate online and secure questionnaires on your current quality of life (one is a general assessment, one is an endometriosis specific assessment of your quality of life and the other is measuring your fatigue levels).
- Daily online diary (2-3 minutes per day): Similar to the screening phase, you will be asked to fill in a daily online diary for the three months of your active treatment (the time when you take the capsules) as well as for four weeks after your finished taking the capsules. This diary captures your pain levels, fatigue levels and the use of any rescue medication (such as ibuprofen). This can be done on your smartphone, tablet or computer.
- Phone call (5-10 minutes) after two weeks: the research team will call you to check on your progress with filling out your daily diary and to monitor for any side effects that you may have experienced after taking the study medication for 2 weeks.
- Midpoint (5-10 minutes): we will ask you to return 6-weeks' worth of medication in a reply-paid postage that will be included in the initial batch of medications we sent out to your home address. We will also schedule a call with you to check on how you're progressing throughout the study, including if there have been any changes in your medical history as well as organise for another batch of study medication to be delivered to your home address.
- End of treatment (30 minutes): this visit will run almost exactly the same as the midpoint visit but with the addition of asking you to have a blood test taken for safety checks (your blood count, liver and kidney function), complete a participant satisfaction questionnaire and several quality of life measures but we will not be sending you any additional medication as you would have finished the treatment period for the study.
- Post-treatment follow-up (5-10 minutes): this will just be a short phone call to check if you experienced any delayed side effects after finishing the treatment period for the study, the completeness of your daily pain diary and ask you to complete two separate quality of life questionnaires.

How much of my time will I need to give?

The total time you will need to give in this study is approximately 8-9 hours (not including travel time to pathology centre) that is spread across 5 months.

What kind of treatment will I receive?

Gynoclear™ is a modification of Gui Zhi Fu Ling Wan and consists of six herbs (Gui Zhi [*Ramulus Cinnamomi*], Fu Ling [*Poria Cocos*], Mu Dan Pi [*Cortex Moutan Radicis*], Bai Shao [*Radix Paeoniae Alba*], Hong Hua [*Flos Carthami*] and Dan Shen [*Salvia miltiorrhiza]* . This is called the 'active treatment'. These ingredients have been traditionally used for gynaecological disorders for hundreds

of years, however we don't know how well this specific combination of ingredients might work in the treatment of endometriosis related pain.

We will be testing this treatment against a study medication that smells and tastes the same but doesn't have any of the active ingredients (called the 'placebo treatment').

Whether you get the active or placebo treatment is based on randomisation. This means the research team don't know in advance which treatment you will get and your allocation to a treatment group is based purely on chance. There is a fifty-fifty chance (50%) that you will receive the active treatment or the placebo treatment.

If you received the placebo treatment, after you have completed your final post-treatment follow-up measures (one month after you stop taking the capsules) you will be offered a complementary three month supply of Gynoclear. This will give you the same treatment as those that were allocated to the active group. You will not be required to fill in any more diaries etc while taking this, and it is completely voluntary if you would like to take this or not.

What benefits will I, and/or the broader community, receive for participating?

If you are currently suffering from endometriosis related chronic pelvic pain you may get a reduction in either your pelvic pain or fatigue from taking the study medication, although there is no guarantee this will happen. You will also get a blood test for safety monitoring (i.e. to ensure there is no pre-existing and previously non-identified issues with your liver and kidney functions). If the study medication is found to be more effective, compared to placebo, for the reduction in endometriosis related pain, this has the potential to benefit women with endometriosis who do not currently experience adequate pain control, or cannot tolerate the side effects of other medications.

Will the study involve any risk or discomfort for me? If so, what will be done to rectify it?

The modified Chinese herbal medicine used in this study is considered safe for oral consumption. The expected adverse event profile of Gynoclear is a combination of adverse events reported for its components, namely: rash or allergic skin reactions and gastrointestinal discomfort. Side effects reported in previous clinical trials also include drowsiness, dizziness and thrombocytopenia, however, it is not known if these effects were due to the ingredient, *Salvia Miltiorrhiza* (Red Sage), that was tested or other drugs. The expected rate for each of these adverse events is infrequent and the expected severity is mild to moderate. Gynoclear is considered relatively safe with no drug related serious adverse events expected at the prescribed dose. There is case of hypoglycemic seizure reported for a patient using cassia cinnamon, but it is unclear if cassia cinnamon caused this event. There is also some concern that cassia cinnamon may cause liver damage due to its coumarin content. However, most evidence suggests that the amount of coumarin in cassia cinnamon is too little to cause adverse effects in most patients.

To monitor for side effects, study staff will monitor you through follow up phone calls two weeks after you start taking the medication as well as mid-way and at the end of the treatment period. We also will take another blood test at the end of the trial to compare to your blood results from before you started the medication. Lastly, we will also check in on you via telephone call 1 month after you stop taking the study medication to make sure you haven't had any delayed reactions.

If you have any side effects that you are concerned about at any time, if you think they are mild please contact the study staff via the contact details in your online diary or at the end of this sheet. If you feel they are serious or life threatening please immediately seek medical attention from your GP or emergency department.

How do you intend to publish or disseminate the results?

We anticipate that the results of this research project will be published and/or presented in a variety of forums. In any publication and/or presentation, we will present information in such a way that a participant can't be identified, such as tables and graphs showing the overall information.

We will be providing an easy to read summary of our results to all major Endometriosis support organisations in Australia, which they can provide to their members/followers.

If you would like to receive a summary of research results individually, please tick the box on the Consent Form.

Will the data and information that I have provided be disposed of?

Please be assured that only the researchers will have access to the raw data you provide. However, your data may be used in other related projects for an extended period of time.

In accordance with the Australian privacy and other relevant laws, you have the right to request access to your information that we have collected and stored by the research team. Please contact the chief investigator, Dr Mike Armour, m.armour@westernsydney.edu.au, 0415 363 201, if you would like access to your information.

We will store all information collected for this study securely and destroy it 15 years after the results are published in accordance with university policy and the Australian Code for the Responsible Conduct of Research.

Can I withdraw from the study?

Participation is entirely voluntary, and you are not obliged to be involved. If you do participate you can withdraw at any time.

If you choose to withdraw, the study investigator or other staff may ask you for your reason for withdrawing to ensure we follow up on any unresolved issues. If you withdraw, you can advise us that you do not consent for us to use the data we collected up until your withdrawal. We will only use the information that you give consent for us to use.

Whatever your decision, it will not affect your medical treatment or your relationship with the medical or other staff involved in the study.

Can I tell other people about the study?

Yes, you can tell other people about the study by providing them with Mahmoud Al-Dabbas's (Clinical Trial Officer) details. They can then contact Mahmoud Al-Dabbas to discuss their participation in the research project and obtain a copy of the information sheet.

What if I require further information?

Please contact Mahmoud Al-Dabbas (Clinical Trial Officer) should you wish to discuss the research further before deciding whether or not to participate.

Mahmoud Al-Dabbas, Clinical Trial Officer, email m.al-dabbas@westernsydney.edu.au or mobile +612 414 357 363.

What if I have a complaint?

If you have any complaints or reservations about the ethical conduct of this research, you may contact the Ethics Committee through Research Engagement, Development and Innovation (REDI) on Tel +61 2 4736 0229 or email humanethics@westernsydney.edu.au.

Any issues you raise will be treated in confidence and investigated fully, and you will be informed of the outcome.

If you agree to participate in this study, you may be asked to sign the Participant Consent Form. The information sheet is for you to keep and the consent form is retained by the researcher/s.

This study has been approved by the Western Sydney University Human Research Ethics Committee. The Approval number is H13256.

WESTERN SYDNEY
UNIVERSITY

Consent Form

Project Title: A placebo controlled, double blind, randomised controlled trial of a modified Gui Zhi Fu Ling Wan formulation (Gynoclear^{TM}) for the treatment of endometriosis.

I hereby consent to participate in the above-named research project.

I acknowledge that:

- I have read the participant information sheet (or where appropriate, have had it read to me) and have been given the opportunity to discuss the information and my involvement in the project with the research team.
- The procedures required for the project and the time involved have been explained to me, and any questions I have about the project have been answered to my satisfaction.

I consent to:

☐ *Providing data such as my age and relevant medical history.*

☐ *Providing daily information about my health in an online diary.*

☐ *Having a blood test taken at the start and end of the study.*

☐ *Filling out a participant satisfaction survey at the end of the study.*

☐ *Filling out quality of life questionnaires.*

I consent for my data and information provided to be used in this project and other related projects for an extended period of time.

Participation is entirely voluntary and you are not obliged to be involved. If you do participate you can withdraw at any time without giving reason.

I understand:

- that my involvement is confidential and that the information gained during the study may be published and stored for other research use but no information about me will be used in any way that reveals my identity but will only be used after additional ethical review.
- that I can withdraw from the study at any time without affecting my relationship with the researcher/s, and any organisations involved, now or in the future.

I would like to receive a summary of the study results when they are available.

Please tick: ☐Yes ☐ No

Participant to sign:

Signed: ______________________________

Name: ______________________________

Date: ______________________________

Researcher obtaining consent to sign:

Signed: ______________________________

Name: ______________________________

Date: ______________________________

This study has been approved by the Human Research Ethics Committee at Western Sydney University. The ethics reference number is: H13256.

What if I have a complaint?

If you have any complaints or reservations about the ethical conduct of this research, you may contact the Ethics Committee through Research Engagement, Development and Innovation (REDI) on Tel +61 2 4736 0229 or email humanethics@westernsydney.edu.au.

Any issues you raise will be treated in confidence and investigated fully, and you will be informed of the outcome.
